# Editorial

**Published:** 1871-01

**Authors:** 


### Editorial.

***January, 1871.***

With this No. commences the *Twenty-Eighth* Annual Volume of this MEDICAL JOURNAL.

In looking over its files, we are proud to discover that although its editorial conduct has from time to time been committed to various hands, it has ever been ranged on the side of high-toned, legitimate medicine, has ever sustained that policy deemed best for its advancement, has condemned and attacked the little, the mean and the sordid, under whatever guise they have tried to come under the wing of the profession—it has ever been ready to recognize true progress and discovery, whilst maintaining that staunch conservatism which, previous to reception, compels innovation to prove itself reform.

Under its present management, the JOURNAL will remain independent of all trammels, of all cliques, private interests or narrow views. It will remain the exponent of the free and enlightened opinions of the profession, to whom its pages will ever be freely open for the discussion and development of the truths of medical science.

Good faith in correspondents, and honorable candor in controversy, will ensure the largest liberty in the use of our columns.

We have no pet notions to foster, or beloved dogmas to disseminate, and had rather, by far, expose ourselves to the charge of inconsistency, than to labor with those who attempt to barricade the advance of modern science by piling up the wrecks and *debris* of the exploded "systems" of olden time.

Exact observation, careful experiment, acute perception, sound judgment—these are what are needed by each individual physician, and then let the Medical Journal make the results, by publication, common property. As yet the material is somewhat chaotic, but the architects and master-builders will, in the not distant future, afford us a temple of Æsculapius, sufficiently approximate to completion to reward the zealous labors of the workmen.

To our contemporaries in journalism, we extend thanks for the valuable "scissorings" they have afforded us in the past; to our correspondents we acknowledge indebtedness for their contributions; to our subscribers we express obligations for their generous support; to our Publishers, Messrs. W. B. KEEN & COOKE, we are grateful for the elegant dress they have given the JOURNAL, for the promptness of its issue, and the invariable courtesy which has characterized their personal association.

To each and all, we heartily tender the best wishes inspired by the advent of the NEW YEAR.

### *Board of Health.*

It is to be hoped that the legislature about to convene at Springfield will reduce the present cumbrous machinery of this institution to a degree of simplicity consistent with a direct, speedy and economical performance of its work. Amidst all of the wheels within wheels (or rings within rings) which constitute what is called the city government, there is probably not one which turns so slowly, with so much friction, consuming so much and generating so little power, and carrying so much dead wood, as this same Board of Health.

A central power to which shall be entrusted the preservation of public health, is essential in every city, but pre-eminently so in Chicago, whose peculiarities of situation, soil, climate and population, render her so especially liable to disease, both endemic and epidemic.

The movement which initiated the idea of a Board of Health for this city was good, as were likewise the plans originally submitted to the legislature for the organization of the department, and had intermeddlers withheld their clumsy fingers from attempts to reform (deform?) it, we should have had at this time, in all probability, an efficient health department, which would have been creditable to its originators, beneficial to the city, acceptable to citizens, and an aid to science, instead of one which is the negative to all these characteristics, and indeed seems to subserve no other purpose than to provide employment for a certain number of very worthy citizens, and to enable one enterprising gentleman (the scavenger) to fatten hogs.

The largest single item of expenditure is that paid to one individual for hauling food to his own pigs, for which purpose householders are required, under penalty, for neglect, to ornament their sidewalks with pails of garbage until such time as it may be convenient to the contractor to gather it.

It is not to be denied that the Board of Health has accomplished some good, for it would be difficult for thirty or forty individuals supposed to be devoted to the attainment of some end, to fail utterly in effecting something, even though infinitessimal. But that it has fulfilled a reasonable proportion of the expectations of its friends and the public, let the public odium, for which it has exchanged its original prestige and popularity, answer.

Passing over its failure as a civic institution, and considering its relations to science, as an accumulator of vital statistics, we ask for data, it furnishes "reports;" for facts, it supplies "opinions;" dissertations on civil engineering by authorities who don't know a transit from a theodolite, and upon hygiene from others ignorant of the relative values of ozone and sulphuretted hydrogen.

The Health Department comprises sufficient ability, energy and honesty (to say nothing of other qualifications) to produce valuable results, if properly utilized, but the radical defects in the system are such that it would seem impossible to evolve order out of chaos without essential changes in the organic law, for which the legislature alone is competent. W. H.

***Malt Extract***

Is the text of a dissertation by the associate editor of the Pharmacist, upon one of the "*Scientific Specialties*" of this prolific era, in which he takes occasion to denounce the deception practiced in the sale, under the above fascinating title of "Lager Beer" (and very poor lager too), at "only six times its retail price." Mr. Ebert's Teutonic antecedents constitute him a better judge of his national beverage than we can pretend to be "*hine illæ lachrymæ*" who, in our ignorance, pronounced the article when presented for our inspection, very bad lager. Who the "most eminent medical authorities in Europe and this country" may be, who agree that this new tonic is the best *dietetic* and *healing remedy* known to modern science, we are not informed, and think it not unlikely that there might be found those who, mistaking *notoriety* for *eminence*, would be willing to purchase the former by the barter of their (good?) names therefor. To those interested in malt extracts, we commend Mr. Ebert's article as containing about all that is worth knowing thereanent; and in discussing that portion of the subject we must take occasion to thank him for the straightforward rebuke administered to those physicians by whose aid, through their signatures to certificates and prescriptions, these nostrum-vendors are sustained. Nor is it to "malt extracts" alone that we refer, but to the whole category of "elixirs," "syrups," "medicated wines, etc.," simple and compound, which are unofficinal and also unreliable. In fact, nothing more nor less than the old curse of patent medicine under a new dress, the threadbare garment of respectability through whose "looped and windowed raggedness" the spectre of quackery is revealed.

With all due courtesy we ask the medical gentlemen, some of whom we could name if required, who specially designate in their prescriptions, the "elixir" or "syrups," etc., of some particular manufacturer, what guarantee they have of the genuineness of the article, the fidelity in preparation, or even, sometimes, acquaintance with its *reputed* formula? They must certainly admit the charge of inconsistency, in that, while careful to avoid patronizing or recommending pharmacists of doubtful repute, they prescribe preparations of whose character their only certificate is the label of a manufacturer personally unknown, backed by the eloquence of the elegant drummer who "*would be pleased to leave a sample box of our preparations*" in your office, and who as a nuisance is only exceeded by the individual, male or female, who is "soliciting subscriptions for an illustrated life of" the Lord knows who. Moreover, the prescription or recommendation to patients of these nostrums, amounts virtually to an abandonment, on the part of the physician, of his own claims to superior skill in diagnosis and judgment in the application of remedies, and a concession thereof to the manufacturing druggist.

Gentlemen, be consistent, make your own formulæ, use remedial agents whose effects you understand, or else include in your category of therapeutical authorities, Helmbold, Radway, Mrs. Winslow, and the rest, and specify in your prescriptions, Ext. of Buchu, as well as Elixir Phosphate, Iron, Quinine and Strychnia, Ready Relief and R. R. R., along with Syrup Chloral Hydrate, and Soothing Syrup with Elixir Valerianate of Ammonia, Indian Cholagogue with Ferro-Phosphorated Elixir of Calisaya Bark, and so *ad infinitum;* and, while earnest students are endeavoring to accumulate reliable data upon which to construct a true science of therapeutics (if that be possible), do not thus encourage in your own minds the growth of empiricism, and foster in others gross quackery. W. H.

### *Alumni Meeting.*

The Alumni of Rush Medical College will bear in mind, that their next annual meeting occurs on the day of the ensuing Commencement Exercises of the College, Wednesday, Feb. 1st., ult. The hour of meeting is 10 A. M., and the place, the lower lecture room of the college. The annual address will be delivered by the President, ABNER HARD, M.D., of Aurora. Business of great importance is to be brought before the Society.

### *Rush Medical College — Spring Course of Instruction.*

Announcement of the usual Spring Course of Instruction will be made soon.

